# Supplementary material for: Fall armyworm infestation, maize production and nutrition security: Evidence from Uganda
Source: PLoS One. 2025 Dec 4;20(12):e0336785. doi: 10.1371/journal.pone.0336785 (PMC12677496; doi:10.1371/journal.pone.0336785)
Supplement: S1 Appendix — (DOCX) [file pone.0336785.s001.docx]

**Appendix Tables and Figures**

**Table A1: Summary statistics of plot, household characteristics and farm management characteristics**

| **Variables** | **Level of FAW Damage** | | |  | | |  |
| --- | --- | --- | --- | --- | --- | --- | --- |
|  | (0)  No damage | (1)  1=Low damage | (2)  2=High damage | (3)  Full sample | (4)  Diff (1)-(0) | (5)  Diff (2)-(0) | (6)  Obs |
| *Plot characteristics* |  |  |  |  |  |  |  |
| Good plot fertility (1/0) | 0.43 | 0.31 | 0.29 | 0.32 | -0.12*** | -0.15*** | 2,157 |
| Gentle slope plot (1/0) | 0.59 | 0.53 | 0.48 | 0.52 | -0.06** | -0.10*** | 2,157 |
| Medium depth soil (1/0) | 0.36 | 0.41 | 0.51 | 0.44 | 0.05* | 0.15*** | 2,157 |
| Irrigation (1/0) | 0.02 | 0.01 | 0.01 | 0.01 | -0.01 | -0.01 | 2,157 |
| Manure use (1/0) | 0.04 | 0.02 | 0.03 | 0.03 | 0.01 | 0.01 | 2,157 |
| Plot tenure (1 = owned, 0 otherwise) | 0.76 | 0.76 | 0.79 | 0.77 | 0.00 | -0.03 | 2,157 |
| Intercropping (1/0) | 0.48 | 0.48 | 0.54 | 0.50 | 0.00 | 0.06** | 2,157 |
| Distance home to plot (minutes) | 23.43 | 23.15 | 21.63 | 22.59 | 0.28 | 1.80 | 2,157 |
| *Household characteristics* |  |  |  |  |  |  |  |
| Sex of hh head (1 = female) | 0.16 | 0.13 | 0.13 | 0.13 | -0.03 | -0.02 | 1,715 |
| Age of the household head (years) | 48.99 | 48.86 | 47.54 | 48.28 | -0.14 | -1.46 | 1,729 |
| Family size (number) | 6.51 | 7.11 | 7.00 | 6.96 | 0.61*** | 0.49*** | 1,729 |
| Education of household head (years) | 9.88 | 8.83 | 9.17 | 9.17 | -1.04*** | -0.71** | 1,729 |
| Value of livestock owned ‘000 UGX | 808.22 | 1055.13 | 1098.70 | 1032.61 | 246.91** | 290.47** | 1,729 |
| Distance to agric extension services (minutes) | 79.08 | 135.70 | 125.93 | 129.57 | 56.61*** | 64.87*** | 1,729 |
| Labour to land ratio | 9.02 | 9.98 | 9.45 | 9.59 | 0.963 | 0.431 | 1,729 |
| *Farm management characteristics* | | | |  |  |  |  |
| Urea rate (kg/Ha) | 8.72 | 8.65 | 6.44 | 7.73 | -0.07 | -2.28 | 2,157 |
| DAP rate (kg/Ha) | 10.70 | 10.54 | 7.55 | 9.30 | -0.15 | -7.55* | 2,157 |
| Seed rate (Kg/Ha) | 28.56 | 28.79 | 30.51 | 29.16 | 0.23 | 1.95 | 2,157 |
| Hired labour (000 UGX/Ha) | 128 | 92 | 63 | 88 | -36*** | -65*** | 2,157 |
| Herbicide use (1/0) | 0.07 | 0.09 | 0.04 | 0.06 | 0.02 | -0.03** | 2,157 |

*, **, *** significant at 1%, 5% and 10%

**Table A2: Marginal Effects of Multinomial Logit Model of FAW Intensity (full results)**

|  | (1) | (2) |
| --- | --- | --- |
|  | Low FAW intensity | High FAW intensity |
| Plot characteristics |  |  |
| Good plot fertility | 0.02 | -0.06 |
|  | (0.046) | (0.046) |
| Gentle slope plot | 0.04 | -0.03 |
|  | (0.037) | (0.037) |
| Tenure | 0.03 | -0.04 |
|  | (0.038) | (0.039) |
| Ln Distance-home to plot | 0.03** | -0.016 |
|  | (0.038) | (0.013) |
| Household characteristics |  |  |
| Female headed | 0.02 | -0.00 |
|  | (0.041) | (0.041) |
| Age | 0.002* | -0.001 |
|  | (0.001) | (0.001) |
| Family size | 0.008 | -0.004 |
|  | (0.006) | (0.006) |
| Education | -0.002 | 0.001 |
|  | (0.003) | (0.003) |
| Ln Value of livestock | -0.001 | 0.013** |
|  | (0.007) | (0.007) |
| Ln Distance (home to agricultural services) | -0.004 | 0.019* |
|  | (0.009) | (0.010) |
| Off-farm income (1/0) | -0.015 | 0.014 |
|  | (0.029) | (0.029) |
| Labor to land ratio | -0.0002 | -0.0001 |
|  | (0.001) | (0.001) |
| Farm management characteristics |  |  |
| Ln Urea (Kg/Ha) | 0.029** | -0.029** |
|  | (0.012) | (0.014) |
| Ln DAP (Kg/Ha) | 0.005 | -0.009 |
|  | (0.011) | (0.014) |
| Ln Seed (Kg/Ha) | -0.036* | 0.041** |
|  | (0.023) | (0.023) |
| Ln hired labor (UGX/Ha) | 0.003* | -0.008*** |
|  | (0.002) | (0.002) |
| Herbicides use | 0.143** | -0.146** |
|  | (0.062) | (0.061) |
| Manure use | -0.048 | -0.025 |
|  | (0.074) | (0.068) |
| Intercropping | -0.030 | 0.050** |
|  | (0.025) | (0.025) |
| Ln insecticide use rate | 0.023 | 0.011 |
|  | (0.027) | (0.03) |
| Instrumental variable:  Risk of FAW | 0.182** | 0.493*** |
|  | (0.099) | (0.104) |
| Village fixed effects | -0.0002 | 0.0004** |
|  | (0.0002) | (0.0001) |
| Seasonal dummy | -0.039** | 0.028 |
|  | (0.019) | (0.017) |
| Mundlak fixed effects |  |  |
| Mean Good plot fertility | -0.038 | 0.021 |
|  | (0.057) | (0.056) |
| Mean Gentle slope plot | -0.006 | -0.031 |
|  | (0.046) | (0.047) |
| Mean Shallow depth plot | 0.028 | 0.008 |
|  | (0.040) | (0.041) |
| Mean Irrigated | 0.231 | -0.321 |
|  | (0.224) | (0.227) |
| Mean Distance-home to plot | -0.0008 | 0.0004 |
|  | (0.0007) | (0.0007) |
| Mean Tenure | -0.054 | -0.018 |
|  | (0.056) | (0.059) |
| Pseudo R2 | 0.129 | 0.129 |
| Observations | 1,960 | 1,960 |

*, **, *** significant at 1%, 5% and 10%; standard errors in parentheses; standard errors clustered at household level

**Table A3: OLS results for the Impact of high and low FAW intensity on maize yield, insecticide-use and DDS for mothers and DDS for children (full results)**

|  | (1) | (2) | (3) | (4) | (5) | (6) | (7) | (8) |
| --- | --- | --- | --- | --- | --- | --- | --- | --- |
| VARIABLES | Maize yield (Kg/Ha) | Maize yield (Kg/Ha) | Insecticide-use (liters/Ha | Insecticide-use (liters/Ha | Children DDS | Children DDS | Mothers DDS | Mothers DDS |
|  |  |  |  |  |  |  |  |  |
| Low FAW intensity | -163.174 | - | 0.170** | - | -0.228 | - | -0.445 | - |
|  | (106.320) | - | (0.082) | - | (0.296) | - | (0.279) | - |
| High FAW intensity | - | -437.767*** | - | 0.100 | - | -0.566** | - | -0.891*** |
|  | - | (110.324) | - | (0.088) | - | (0.283) | - | (0.274) |
| Good_Plot_fertility | 212.958 | 140.851 | 0.169* | -0.005 | -0.350 | 0.575 | -0.351 | 0.140 |
|  | (162.382) | (171.440) | (0.096) | (0.127) | (0.277) | (0.419) | (0.264) | (0.385) |
| Gentle_slope | -74.487 | -246.241 | 0.073 | -0.038 | -0.293* | 0.269 | -0.260 | 0.173 |
|  | (124.479) | (168.713) | (0.106) | (0.215) | (0.162) | (0.229) | (0.172) | (0.222) |
| Tenure | -72.307 | 87.371 | -0.066 | 0.275* | 0.085 | -0.048 | 0.118 | 0.084 |
|  | (134.165) | (137.029) | (0.101) | (0.160) | (0.190) | (0.232) | (0.173) | (0.208) |
| lnDistance | -54.930 | -13.987 | 0.004 | 0.035 | 0.038 | 0.015 | 0.032 | 0.068 |
|  | (37.817) | (37.039) | (0.038) | (0.042) | (0.106) | (0.089) | (0.089) | (0.085) |
| femalehead | -195.306 | -236.973** | -0.341*** | -0.224* | -0.119 | -0.136 | -0.131 | -0.128 |
|  | (142.598) | (115.815) | (0.094) | (0.133) | (0.494) | (0.381) | (0.436) | (0.446) |
| age | 2.450 | 3.871 | 0.006** | -0.000 | -0.020 | -0.019* | -0.022* | -0.024** |
|  | (3.907) | (3.015) | (0.003) | (0.004) | (0.013) | (0.010) | (0.013) | (0.010) |
| hh_size | 49.709** | 12.376 | 0.021 | -0.011 | 0.022 | 0.053 | 0.046 | 0.080 |
|  | (21.459) | (18.404) | (0.016) | (0.020) | (0.065) | (0.047) | (0.063) | (0.051) |
| education | -9.252 | -5.067 | -0.010 | -0.001 | 0.054* | 0.027 | 0.083*** | 0.024 |
|  | (9.968) | (9.482) | (0.009) | (0.010) | (0.032) | (0.025) | (0.031) | (0.026) |
| lnValue_livestock | -12.326 | 7.838 | 0.001 | 0.025 | 0.188** | 0.061 | 0.184** | 0.055 |
|  | (21.344) | (19.209) | (0.018) | (0.027) | (0.074) | (0.052) | (0.075) | (0.057) |
| lnDistance_2 | -28.015 | 6.855 | 0.014 | 0.080** | -0.209*** | -0.202*** | -0.168** | -0.207*** |
|  | (28.036) | (31.736) | (0.024) | (0.033) | (0.080) | (0.071) | (0.073) | (0.069) |
| off_farm_hh | -58.899 | -120.174 | 0.132 | -0.086 | -0.085 | 0.566*** | -0.098 | 0.600*** |
|  | (103.259) | (88.098) | (0.093) | (0.099) | (0.270) | (0.212) | (0.259) | (0.208) |
| labour_land | 1.863 | -0.321 |  |  | -0.001 | -0.016 | -0.003 | -0.019 |
|  | (2.880) | (2.351) |  |  | (0.010) | (0.011) | (0.011) | (0.012) |
| s_dummy | -37.949 | -175.966*** | -0.088 | -0.088 |  |  |  |  |
|  | (65.374) | (60.622) | (0.058) | (0.057) |  |  |  |  |
| Good_Plot_fertility_x | -138.435 | -43.706 | -0.247* | -0.106 | 0.331 | -0.872* | 0.260 | -0.557 |
|  | (199.799) | (204.932) | (0.136) | (0.193) | (0.432) | (0.473) | (0.405) | (0.444) |
| Gentle_slope_x | 32.503 | 255.635 | 0.039 | -0.014 | 0.903*** | 0.430 | 0.993*** | 0.448 |
|  | (160.189) | (190.356) | (0.135) | (0.241) | (0.324) | (0.325) | (0.313) | (0.310) |
| shallow_depth_plot_x | -115.397 | -284.881** | -0.143 | -0.186* | -0.402 | 0.373 | -0.095 | 0.451 |
|  | (149.637) | (125.771) | (0.099) | (0.097) | (0.331) | (0.334) | (0.321) | (0.358) |
| irrigated_x | -85.215 | -362.622 | 0.750 | -0.285 | 1.576 | 3.054*** | 0.743 | 2.480*** |
|  | (388.127) | (246.964) | (0.673) | (0.198) | (1.343) | (0.668) | (1.231) | (0.650) |
| Distance_x | 1.726 | -1.812 | 0.000 | -0.001 | 0.001 | 0.001 | 0.005 | 0.002 |
|  | (1.800) | (1.630) | (0.002) | (0.001) | (0.007) | (0.005) | (0.006) | (0.005) |
| Tenure_x | -131.167 | -81.506 | 0.141 | -0.122 | -0.259 | -0.675* | -0.174 | -0.708** |
|  | (190.862) | (186.245) | (0.123) | (0.182) | (0.477) | (0.371) | (0.435) | (0.338) |
| village | -0.593 | 0.406 | -0.000 | 0.000 | 0.000 | -0.000 | 0.000 | -0.000 |
|  | (0.562) | (0.522) | (0.000) | (0.001) | (0.002) | (0.001) | (0.001) | (0.001) |
| lnhh_income_000 |  |  | -0.052 | -0.080* |  |  |  |  |
|  |  |  | (0.036) | (0.047) |  |  |  |  |
| SUPPORT_FAW |  |  | 0.309*** | 0.339*** |  |  |  |  |
|  |  |  | (0.105) | (0.123) |  |  |  |  |
| Constant | 1,935.428*** | 1,681.607*** | 0.162 | 0.573* | 6.518*** | 7.374*** | 6.066*** | 7.659*** |
|  | (360.150) | (303.107) | (0.329) | (0.333) | (0.933) | (0.770) | (0.876) | (0.777) |
|  |  |  |  |  |  |  |  |  |
| Observations | 1,171 | 1,186 | 1,184 | 1,205 | 240 | 273 | 239 | 272 |
| R-squared | 0.029 | 0.054 | 0.059 | 0.048 | 0.182 | 0.213 | 0.224 | 0.250 |

*** p<0.01, ** p<0.05, * p<0.1; Standard errors were clustered at household level.

### **Table A4: Second step regression for Yield (selectivity correction based on multinomial logit)**

|  | (1) | (2) | (2) |
| --- | --- | --- | --- |
|  | Yield | Yield | Yield |
| VARIABLES | *FAW=0* | *FAW=1* | *FAW=2* |
| Good_Plot_fertility | 0.0344 | 0.185 | -0.0445 |
|  | (0.351) | (0.168) | (0.218) |
| Gentle_slope | -0.245 | -0.124 | -0.125 |
|  | (0.322) | (0.149) | (0.191) |
| Tenure | -0.183 | -0.0952 | 0.00755 |
|  | (0.360) | (0.169) | (0.190) |
| lnDistance | -0.125 | -0.112** | 0.00143 |
|  | (0.0774) | (0.0446) | (0.0619) |
| Femalehead | -0.300* | -0.159 | 0.00664 |
|  | (0.180) | (0.115) | (0.105) |
| Age | -0.0103** | -0.00377 | 0.00305 |
|  | (0.00500) | (0.00315) | (0.00378) |
| hh_size | 0.00729 | 0.0118 | -0.00306 |
|  | (0.0283) | (0.0162) | (0.0200) |
| Education | -0.00923 | 0.00227 | -0.000898 |
|  | (0.0116) | (0.00764) | (0.00707) |
| lnValue_livestock | 0.0264 | 0.00543 | 0.0181 |
|  | (0.0282) | (0.0201) | (0.0181) |
| lnDistance_2 | 0.0419 | 0.00199 | 0.0181 |
|  | (0.0418) | (0.0256) | (0.0296) |
| off_farm_hh | 0.0513 | -0.0170 | -0.119 |
|  | (0.131) | (0.0759) | (0.0756) |
| Labour_land | -0.00211 | 0.00363 | 0.000750 |
|  | (0.00597) | (0.00295) | (0.00278) |
| Good_Plot_fertility_x | 0.0387 | -0.0831 | -0.00522 |
|  | (0.367) | (0.186) | (0.252) |
| Gentle_slope_x | -0.0340 | -0.0506 | 0.174 |
|  | (0.352) | (0.158) | (0.197) |
| shallow_depth_plot_x | -0.651** | 0.0889 | 0.0230 |
|  | (0.266) | (0.122) | (0.0989) |
| irrigated_x | -0.270 | -0.0749 | -0.425 |
|  | (0.525) | (0.410) | (0.423) |
| Distance_x | 0.00106 | 0.00372** | -0.00194 |
|  | (0.00231) | (0.00152) | (0.00233) |
| Tenure_x | 0.189 | 0.0559 | 0.0468 |
|  | (0.425) | (0.225) | (0.262) |
| _mo | - | -0.152 | -0.848 |
|  | - | (0.146) | (0.582) |
| _m1 | -2.427** | - | 0.309 |
|  | (1.060) | - | (0.782) |
| _m2 | 2.346** | 0.587** | - |
|  | (1.007) | (0.292) | - |
| Sigma2 | 10.45 | 1.098*** | 0.923 |
|  | (9.342) | (0.351) | (1.469) |
| rho0 | - | -0.186 | -0.446 |
|  | - | (0.160) | (0.382) |
| rho1 | -0.963*** | - | 0.413 |
|  | (0.145) | - | (0.629) |
| rho2 | 0.931*** | 0.718*** | - |
|  | (0.131) | (0.273) | - |
| Constant | 7.680*** | 8.072*** | 6.692*** |
|  | (0.481) | (0.520) | (0.554) |

*** p<0.01, ** p<0.05, * p<0.1; Bootstrapped Standard errors in parentheses

### **Table A5: Second step regression for Sales (selectivity correction based on multinomial logit)**

|  | (1) | (2) | (2) |
| --- | --- | --- | --- |
|  | Sales | Sales | Sales |
|  | $FAW=0$ | $FAW=1$ | $FAW=2$ |
| Good_Plot_fertility | 0.565 | -0.313 | -0.726 |
|  | (0.879) | (0.569) | (0.563) |
| Gentle_slope | -0.566 | 0.184 | 0.236 |
|  | (0.873) | (0.464) | (0.646) |
| Tenure | 0.179 | 0.446 | -0.00228 |
|  | (0.867) | (0.479) | (0.719) |
| lnDistance | -0.0535 | 0.0606 | 0.140 |
|  | (0.262) | (0.161) | (0.263) |
| femalehead | -0.230 | -0.297 | 0.209 |
|  | (0.538) | (0.392) | (0.385) |
| age | -0.0574*** | -0.0206* | 0.0183 |
|  | (0.0201) | (0.0108) | (0.0153) |
| hh_size | 0.0107 | 0.0847 | 0.0643 |
|  | (0.107) | (0.0561) | (0.0723) |
| education | -0.00539 | 0.0348 | 0.00164 |
|  | (0.0459) | (0.0309) | (0.0430) |
| lnValue_livestock | 0.235*** | 0.197*** | 0.151** |
|  | (0.0756) | (0.0674) | (0.0770) |
| lnDistance_2 | 0.0995 | 0.00265 | 0.00263 |
|  | (0.118) | (0.0897) | (0.0938) |
| off_farm_hh | 0.0907 | 0.937*** | -0.696** |
|  | (0.386) | (0.245) | (0.296) |
| Land_labour | -0.0536* | -0.0269** | -0.0336*** |
|  | (0.0284) | (0.0118) | (0.0116) |
| Good_Plot_fertility_x | 0.528 | 0.121 | 0.545 |
|  | (1.027) | (0.642) | (0.674) |
| Gentle_slope_x | 0.342 | -0.209 | -0.00134 |
|  | (0.901) | (0.530) | (0.582) |
| shallow_depth_plot_x | -0.167 | -0.273 | 0.989** |
|  | (0.660) | (0.441) | (0.478) |
| irrigated_x | 0.0228 | -2.202 | -2.197 |
|  | (1.897) | (1.384) | (2.022) |
| Distance_x | 0.00451 | 0.00272 | 0.0127 |
|  | (0.00848) | (0.00670) | (0.00882) |
| Tenure_x | -0.666 | -1.292** | -0.824 |
|  | (1.118) | (0.581) | (0.810) |
| _m0 | - | -1.062* | -3.597** |
|  | - | (0.612) | (1.742) |
| _m1 | -5.776 | - | 3.980 |
|  | (3.763) | - | (2.851) |
| _m2 | 5.705 | 2.193* | - |
|  | (3.469) | (1.206) | - |
| Sigma2 | 64.29 | 13.13* | 32.96 |
|  | (87.72) | (6.924) | (48.06) |
| rho0 | - | -0.376** | -0.804*** |
|  | - | (0.163) | (0.154) |
| rho1 | -0.924*** | - | 0.889*** |
|  | (0.357) | - | (0.339) |
| rho2 | 0.913*** | 0.776*** | - |
|  | (0.283) | (0.290) | - |
| Constant | 6.901*** | 6.714*** | 4.177** |
|  | (1.390) | (1.794) | (1.965) |

*** p<0.01, ** p<0.05, * p<0.1; Bootstrapped Standard errors in parentheses

### **Table A6: Second step regression for Insecticide use (selectivity correction based on multinomial logit)**

|  | (1) | (2) | (3) |
| --- | --- | --- | --- |
|  | Insecticide-use | Insecticide-use | Insecticide-use |
| VARIABLES | $FAW=0$ | $FAW=1$ | $FAW=2$ |
| Good_Plot_fertility | 0.0102 | 0.0908 | 0.00586 |
|  | (0.132) | (0.0730) | (0.0782) |
| Gentle_slope | 0.00534 | -0.0196 | -0.0322 |
|  | (0.145) | (0.0894) | (0.103) |
| Tenure | 0.0359 | -0.0702 | 0.124 |
|  | (0.115) | (0.0777) | (0.0880) |
| lnDistance | -0.0557* | 0.0120 | 0.0173 |
|  | (0.0300) | (0.0243) | (0.0281) |
| femalehead | -0.138* | -0.140*** | -0.103* |
|  | (0.0716) | (0.0497) | (0.0549) |
| age | -0.00250 | 0.00351** | 0.000598 |
|  | (0.00233) | (0.00157) | (0.00202) |
| hh_size | -0.00522 | 0.0162** | -0.00207 |
|  | (0.0140) | (0.00811) | (0.0102) |
| education | 0.000791 | -0.00497 | -0.000637 |
|  | (0.00540) | (0.00381) | (0.00416) |
| lnValue_livestock | 0.0176 | 0.00282 | 0.00891 |
|  | (0.0126) | (0.00757) | (0.0123) |
| lnDistance_2 | 0.0157 | 0.00304 | 0.0381*** |
|  | (0.0218) | (0.0110) | (0.0139) |
| off_farm_hh | 0.0534 | 0.0588 | -0.0427 |
|  | (0.0674) | (0.0400) | (0.0382) |
| Land_labour | -0.00242 | -0.000510 | -0.00108 |
|  | (0.00266) | (0.00257) | (0.00230) |
| Good_Plot_fertility_x | -0.0173 | -0.128 | -0.0753 |
|  | (0.141) | (0.0886) | (0.0985) |
| Gentle_slope_x | -0.0222 | 0.0942 | -0.0302 |
|  | (0.151) | (0.101) | (0.111) |
| shallow_depth_plot_x | -0.130 | -0.0728* | -0.0691 |
|  | (0.0947) | (0.0435) | (0.0477) |
| irrigated_x | -0.0136 | 0.396 | -0.234** |
|  | (0.268) | (0.309) | (0.105) |
| Distance_x | 0.00123 | 0.000136 | -2.00e-05 |
|  | (0.00110) | (0.000979) | (0.000963) |
| Tenure_x | 0.0420 | 0.0795 | -0.0494 |
|  | (0.150) | (0.105) | (0.118) |
| _m0 | - | -0.0474 | -0.175 |
|  | - | (0.0822) | (0.202) |
| _m1 | -1.173*** | - | 0.206 |
|  | (0.440) | - | (0.338) |
| _m2 | 1.109*** | -0.0264 | - |
|  | (0.414) | (0.166) | - |
| Sigma2 | 2.410 | 0.228*** | 0.300 |
|  | (1.590) | (0.0426) | (0.321) |
|  |  |  |  |
| rho0 | - | -0.127 | -0.409 |
|  | - | (0.198) | (0.301) |
| rho1 | -0.969*** | - | 0.484 |
|  | (0.141) | - | (0.518) |
| rho2 | 0.916*** | -0.0710 | - |
|  | (0.137) | (0.393) | - |
| Constant | 0.104 | -0.161 | 0.141 |
|  | (0.203) | (0.258) | (0.243) |

*** p<0.01, ** p<0.05, * p<0.1; Bootstrapped Standard errors in parentheses

### **Table A7: Second step regression for HDDS for children and mothers (selectivity correction based on multinomial logit)**

|  | DDS for children | | |  | DDS for mothers | | |
| --- | --- | --- | --- | --- | --- | --- | --- |
|  | (1) | (2) | (3) |  | (1) | (2) | (2) |
|  | DDS-Child | DDS-Child | DDS-Child |  | DDS-Mothers | DDS-Mothers | DDS-Mothers |
| VARIABLES | $FAW=0$ | $FAW=1$ | $FAW=2$ |  | $FAW=0$ | $FAW=1$ | $FAW=2$ |
|  |  |  |  |  |  |  |  |
| Good_Plot_fertility | 0.807 | -0.287 | 0.106 |  | -0.120 | -0.0285 | 0.103 |
|  | (1.249) | (0.508) | (0.464) |  | (0.888) | (0.486) | (0.418) |
| Gentle_slope | 0.236 | -0.134 | 0.452 |  | 0.145 | 0.0213 | 0.409 |
|  | (0.879) | (0.457) | (0.408) |  | (0.847) | (0.434) | (0.434) |
| Tenure | -0.480 | 0.372 | -0.162 |  | -0.299 | 0.464 | -0.231 |
|  | (0.618) | (0.488) | (0.376) |  | (0.665) | (0.480) | (0.386) |
| lnDistance | -0.153 | 0.378*** | -0.0556 |  | -0.0527 | 0.410** | -0.0145 |
|  | (0.208) | (0.141) | (0.171) |  | (0.241) | (0.167) | (0.158) |
| femalehead | 0.633 | 0.472 | -0.363 |  | 0.739 | 0.315 | -0.229 |
|  | (1.419) | (0.375) | (0.409) |  | (1.061) | (0.347) | (0.385) |
| age | 0.0270 | -0.0107 | -0.0109 |  | 0.0126 | -0.00894 | -0.0118 |
|  | (0.0254) | (0.0124) | (0.0113) |  | (0.0209) | (0.0116) | (0.0135) |
| hh_size | -0.120 | 0.0954* | 0.00516 |  | -0.0308 | 0.0693 | 0.0342 |
|  | (0.122) | (0.0546) | (0.0493) |  | (0.109) | (0.0523) | (0.0455) |
| education | 0.179*** | -0.00858 | 0.0181 |  | 0.222*** | 0.0284 | -0.00111 |
|  | (0.0604) | (0.0308) | (0.0231) |  | (0.0571) | (0.0285) | (0.0267) |
| lnValue_livestock | -0.0667 | 0.136* | -0.0963 |  | -0.112 | 0.0944 | -0.102 |
|  | (0.115) | (0.0748) | (0.0712) |  | (0.0981) | (0.0660) | (0.0820) |
| lnDistance_2 | -0.379*** | -0.0752 | -0.238** |  | -0.347*** | -0.105 | -0.288*** |
|  | (0.140) | (0.0760) | (0.0960) |  | (0.126) | (0.0750) | (0.0991) |
| lnprice | 0.685 | 0.349 | -0.0719 |  | 0.632 | 0.229 | -0.509** |
|  | (0.673) | (0.353) | (0.258) |  | (0.694) | (0.244) | (0.231) |
| lnNu_animals | 0.989*** | 0.375** | 0.563*** |  | 0.922*** | 0.440*** | 0.653*** |
|  | (0.336) | (0.156) | (0.142) |  | (0.262) | (0.131) | (0.159) |
| off_farm_hh | 0.338 | -0.153 | 0.718*** |  | 0.396 | -0.272 | 0.788*** |
|  | (0.438) | (0.252) | (0.202) |  | (0.340) | (0.231) | (0.194) |
| Labour land | 0.0208 | 0.00145 | -0.0176 |  | 0.0165 | 0.00193 | -0.0189 |
|  | (0.0150) | (0.00998) | (0.0131) |  | (0.0161) | (0.0110) | (0.0118) |
| Good_Plot_fertility_x | 0.0635 | -0.187 | -0.374 |  | 0.607 | -0.414 | -0.354 |
|  | (1.452) | (0.542) | (0.555) |  | (0.957) | (0.541) | (0.504) |
| Gentle_slope_x | 0.872 | 0.482 | -0.0282 |  | 0.890 | 0.463 | -0.0132 |
|  | (0.957) | (0.520) | (0.410) |  | (0.973) | (0.492) | (0.412) |
| shallow_depth_plot_x | -0.296 | 0.238 | -0.125 |  | 0.0869 | 0.239 | 0.0315 |
|  | (1.150) | (0.357) | (0.300) |  | (1.256) | (0.324) | (0.319) |
| irrigated_x | 1.430 | 2.117* | 1.790 |  | 1.338 | 2.472** | 2.044* |
|  | (2.306) | (1.222) | (1.097) |  | (1.658) | (1.212) | (1.071) |
| Distance_x | 0.0184** | -0.0167** | 0.00184 |  | 0.0186*** | -0.0139* | 0.00456 |
|  | (0.00735) | (0.00733) | (0.00498) |  | (0.00685) | (0.00769) | (0.00553) |
| Tenure_x | 0.526 | -0.900 | -0.322 |  | 0.374 | -0.627 | -0.277 |
|  | (1.030) | (0.671) | (0.563) |  | (0.975) | (0.646) | (0.488) |
| _m0 | - | -0.569 | 1.754 |  | - | -0.151 | 1.467 |
|  | - | (0.708) | (1.241) |  | - | (0.642) | (1.188) |
| _m1 | 1.569 | - | -1.882 |  | 3.049 | - | -1.467 |
|  | (3.904) | - | (2.031) |  | (3.207) | - | (1.875) |
| _m2 | -2.241 | -0.899 | - |  | -3.729 | -1.095 | - |
|  | (3.619) | (1.152) | - |  | (3.001) | (1.268) | - |
| Sigma2 | 7.890 | 7.890 | 6.699 |  | 20.93 | 3.142 | 4.990 |
|  | (44.44) | (55.49) | (16.57) |  | (38.60) | (3.568) | (8.983) |
| rho0 | - | -0.408 | 0.869** |  | - | -0.109 | 0.843** |
|  | - | (0.412) | (0.351) |  | - | (0.368) | (0.401) |
| rho1 | 0.717 | - | -0.932 |  | 0.855 | - | -0.842 |
|  | (0.873) | - | (0.582) |  | (0.783) | - | (0.654) |
| rho2 | -1.023 | -0.646 | - |  | -1.046* | -0.793 | - |
|  | (0.787) | (0.613) | - |  | (0.609) | (0.613) | - |
| Constant | -2.723 | 0.578 | 7.009** |  | -2.294 | 1.178 | 9.830*** |
|  | (4.325) | (2.904) | (2.854) |  | (4.303) | (2.564) | (2.392) |

*** p<0.01, ** p<0.05, * p<0.1; Bootstrapped Standard errors in parentheses

**Table A8: Estimates of Yield Losses from FAW infestation from Literature**

| **Literature source** | **Countries** | **Year of study** | **Estimated mean yield loss from FAW infestation** |
| --- | --- | --- | --- |
| Day et al, 2017 | Benin, Cameroon, DRC, Ethiopia, Ghana, Malawi, Mozambique, Nigeria , Uganda, Tanzania, Zambia, Zimbabwe | 2017 | 0.42 |
| Rwomushana et al, 2018 | Zambia | 2018 | 0.35 |
| Baudron et al, 2019 | Zimbabwe | 2019 | 0.12 |
| De groote et al, 2020 | Kenya | 2020 | 0.33 |
| Kassie et al, 2020 | Ethiopia | 2019 | 0.12 |
| Chimweta et al, 2019 | Zimbabwe | 2019 | 0.58 |
| **Mean from literature** | **All** | **all** | **0.31** |
