# Supplementary material for: Fall armyworm infestation, maize production and nutrition security: Evidence from Uganda
Source: PLoS One. 2025 Dec 4;20(12):e0336785. doi: 10.1371/journal.pone.0336785 (PMC12677496; doi:10.1371/journal.pone.0336785)
Supplement: S2 Appendix — (DOCX) [file pone.0336785.s002.docx]

Reduces maize availability

FAW infestation

Reduces maize Yields

Reduces access to diverse foods

Reduces maize sales

Less income to purchase diverse foods

Reduces DDS (Food nutrition security)

Increases expenditure on pesticides and labour

Net maize sellers

Rural food markets

**Figure A1: Pathways for the Impact of FAW on Food Nutrition Security**

**Figure A2: Kernel density plots of log of maize yield and log of maize sales by FAW Intensity**

**Figure A3: Kernel density plots of log of insecticide-use-rate by FAW Intensity**
