# Supplementary material for: Fall armyworm infestation, maize production and nutrition security: Evidence from Uganda
Source: PLoS One. 2025 Dec 4;20(12):e0336785. doi: 10.1371/journal.pone.0336785 (PMC12677496; doi:10.1371/journal.pone.0336785)
Supplement: S1 Questionnaire — (DOCX) [file pone.0336785.s004.docx]

Questionnaire

International Center of Insect Physiology and Ecology (*icipe*)

Baseline Survey Report for Smallholder Maize Farmers in Kamuli District, Uganda, 2019

Introductory and consent statement:

“Dear Sir/Madam, I work for the International Center of Insect Physiology and Ecology (*icipe*). We are conducting a survey to study to understand importance of three main constraints (i.e. Striga, FAW and stemborer) on maize agricultural production and livelihoods in your village, and to identify the important mechanisms for promoting wide adoption of Push pull technology to manage these constraints. Your household response to these questions would remain anonymous. Taking part in this study is voluntary; you choose not to take part; you have the right not to participate and there will be no consequences.

Do you and your family consent to provide information? 1=Yes, 0=No ………………………….

“Thank you for your kind co-operation”.

MODULE 1. HOUSEHOLD AND VILLAGE IDENTIFICATION

|  |  | / |  |  | / | 2019 |
| --- | --- | --- | --- | --- | --- | --- |

| Household Identification | Code |  | Interview details | | Code |
| --- | --- | --- | --- | --- | --- |
| A1. County: |  |  | A16. Date of interview (dd/mm/yyyy): | |  |
| A2. Subcounty: |  |  | A17. Name of enumerator: | | |
| A3.: Parish |  |  | A18. Name of supervisor: | | |
| A4. Village: |  |  |  | |  |
|  |  |  | GPS reading of homestead | |  |
| A5. Name of the respondent: | |  | A19. Way point number: | |  |
| A6. Sex of the respondent 1=Male; 0=Female0 |  |  | A20. Latitude (North): | |  |
| A7. Name of household head: |  |  | A21. Longitude (East): | |  |
| A8. Sex of household head 1=Male; 0=Female0 |  |  | A22. Altitude (meter above sea level): | |  |
| A9. Name of spouse (only for male-headed hhld): | |  |  |  |  |
| A10. Sex of spouse 1=Male; 0=Female |  |  |  |  |  |
| A11. Cell phone number of the household head: |  |  |  |  |  |
| A12. Cell phone number of spouses: |  |  |  |  |  |
| A.13 Name of farmer group |  |  |  |  |  |
| A.14 Role of farmer in the group (1=leader, 0=member) |  |  |  |  |  |
| A.15 How many years have you been a manner to this group |  |  |  |  |  |

MODULE 2: HOUSEHOLD COMPOSITION AND CHARACTERISTICS (Household Members: Persons who live together and eat together from the same pot (share food), including hired labour, students and spouse living and working in another location but excluding visitors)

Now we would like some information about the people who usually live in your household or who are staying with you now.

| PERSON ID | NAME OF MEMBER  Please give me the names of the persons who usually live in your household | RELATIONSHIP TO THE HOUSE- HOLD HEAD  What is the rela- tionship of (NAME) to the head of the house- hold? *  (See code 1) | Marital status See code 2) | SEX | EDUCATION | | RESIDENCE | |  | AGE | WHAT IS THE MAIN OCCU- PATION | WHAT IS THIS MEMBERS LABOR CONTRIBUTION TO FARMING AC- TIVITIES IN THE  HOUSEHOLD (%)  *THIS SHOULD SUM UP TO 100* |
| --- | --- | --- | --- | --- | --- | --- | --- | --- | --- | --- | --- | --- |
|  |  |  |  | What is the sex of (NAME)  1=male 2=female | Is .......  attending formal school? 1 = Yes  0 = No | What is the highest level of formal edu- cation?  .... com- pleted?  (see CODE 3 below) | Does (NAME)  usually live here? 1=yes 0=No | If no to B5.1 is this mem- ber a guest?  1=yes 0=No | Did (NAME)  stay here last night? 1=yes 0=No | How old is (NAME)? (IN YEARS) |  |  |
| PID | 2A0 | 2A1 | 2A2 | 2A3 | 2A4.1 | 2A4.2 | 2A5.1 | 2A5.2 | 2A5.3 | 2A6 | 2A7 |  |
|  |  |  |  |  |  |  |  |  |  |  |  |  |
|  |  |  |  |  |  |  |  |  |  |  |  |  |
|  |  |  |  |  |  |  |  |  |  |  |  |  |

| CODE 1 | CODE 2 | CODE 3 | CODE 4 | CODE5 |
| --- | --- | --- | --- | --- |
| 1.Household head 2.Spouse 3.Son/daughter 4.Parent  5.Son/daughter-in-law 6.Grandson/granddaughter   1. Other relative 2. Hired worker 3. Other, specify…………… | 1. Married living with spouse 2. Married living without spouse 3.Divorced/separated 4.Widow/widower   5.Never married | 100. Religious education 0=Pre-Primary  1=Primary 1/ Adult education 2=Primary 2  3=Primary 3  4=Primary 4  5=Primary 5  6=Primary 6  7=Primary 7  8=Primary 8  9=Secondary 1 (OL1) or (Jr. I) 10=Secondary 2 (OL2) or (Jr. II) 11=Secondary 3 (OL3)  12=Secondary 4 (OL4)  13=Secondary 5 (AL1)  14=Secondary 6 (AL2)  15=Tertiary 1 | 1. Farming (crop+ livestock) 2. Salaried employment 3.Self-employed off-farm 3. Casual laborer on-farm 4. Casual laborer off-farm 6.School/college child 7.Non-school child 8.Other, specify…………. | 1. Full time 2. Part time 3. Not a worker |

|  |  | 16=Tertiary 2  17=Tertiary 3  18=Tertiary 4  19=Tertiary 5 20=Postgraduate  99= Never in school/illiterate |  |  |
| --- | --- | --- | --- | --- |

MODULE 3: CROP PRODUCTION BY THE HOUSEHOLD DURING (INCLUDE SEASON)

*The farmer should have at least a maize plot or. Sorghum/millet/rice*

3.1A Total owned land (hectares) ………………………………

3.1B list the top five crops grown in this household in order of importance (*codes in annex A*) 1) 2 3) 4) 5)

3.1C Total land cultivated (acres) (own + rented in + borrowed in) in the: July/August 2019 planting season (current) Feb/march 2019 (last season) Aug/sept2018

3.1D Total rented-in land (acres) during: July/August 2019 planting season (current) Feb/march 2019 (last season) Aug/sept2018

3.1E (i) Are you aware of fall armyworm? (1= yes; 0=no)

1. From these pictures can you identify fall armyworm. (enumerator please show the pictures);1= yes;0=no
2. Is fall armyworm a major constraint to maize production on your farm (1= yes; 0=no)

3.1F (i)Are you aware of stem-borer? 1= yes;0=no

1. From these pictures can you identify stem-borer? (enumerator please show the pictures);1= yes;0=no
2. Is stem-borer a major constraint to maize production on your farm (1= yes; 0=no)

3.1G Is Striga a major constraint to maize production on your farm (1= yes; 0=no)

3.1H If yes, how would you rate Striga infestation in your farm (on a scale of 1 to 10, where 10 is indicates highest infestation?

3.1I Total area cultivated for maize (acres) during the July/August 2019 planting season (current season ) Feb/march 2019 planting (last season) Aug/sept2018

3.1J A How many plots of maize did you have?: July/August 2019 planting season (current) Feb/march 2019 (last season) Aug/sept2018

MODULE 3 PART A: MAIZE PLOT INFORMATION IN CROPPING SEASONs { 1=Short rain season (Aug/Sept 2018 planting); 2= Long rain season (Feb/March 2019 plant- ing,3= current season (Aug/Sept 2019 planting))} [ This for ONLY maize plot, but capture intercrops on the plot]

| Serial No |  | Parcel ID (start with one next to residence) | plo t ID | Plot loca- tion name | plo t are a (ac res  ) | plot ten- ure  Cod e 1 | Who in the hhld makes de- cisions on crops to be planted, input use, and tim- ing of cropping activities on this [Sub-  PLOT]?  *CODES 2* | Plot dis- tance to residence (walking minutes) | Intercropping practice | | | | | Percent of area for sec- ond main  crop |
| --- | --- | --- | --- | --- | --- | --- | --- | --- | --- | --- | --- | --- | --- | --- |
|  | Season  1=Short rain season (Aug/Sept 2018 planting)  2=Long rain season (Feb/Marchl 2019 planting)  3=current season (Aug/Sept 2019 planting) |  |  |  |  |  |  |  | Inter- crop- ping on this plot? 0=No  1=Yes | If yes to Col 3A8,  num- ber of crops grown  ? | If yes to col- umn A8, first main crop grown:  Crop code 2 | Per- cent of area for first main crop | if yes col- umn 3A8, second main crop grown: Crop code 3 |  |

COLUMN A8

| 3  A 1 |  | 3A2 | 3A  3 | 3A4 | 3A  5 | 3A6  a | 3A6b | 3A7 | 3A8 | 3A9 | 3A10 | 3A11 | 3A12 | 3A13 |
| --- | --- | --- | --- | --- | --- | --- | --- | --- | --- | --- | --- | --- | --- | --- |
|  |  |  |  |  |  |  |  |  |  |  |  |  |  |  |
|  |  |  |  |  |  |  |  |  |  |  |  |  |  |  |
|  |  |  |  |  |  |  |  |  |  |  |  |  |  |  |

A/ for rented out/shared out and borrowed out plots, please fill up to

| CODE 1 |  | CODE 3 | | | |
| --- | --- | --- | --- | --- | --- |
| 1. Owned 4. Borrowed in 2. 5. Borrowed out Rented/shared 6. Other, specify….. in   Rented/shared out | Code 2 1=head 2=Spouse  3=head and spouse jointly 4=Other household member  5=head and other household member(s) 6=Spouse and other household member(s) 7=head, spouse and other household member(s) | Maize Sorghum Teff Wheat Barley Millet Beans Chickpeas | Groundnut Potato Tomato Cabbage Black Paper Papaya Lentil Linseed Sesame | Garlic desmodium Khat Banana Mango Sugar Cane Coffee Gesho | Eucalyptus Grazing land Fallow land Haricot bean Other crop, spec- ify... |

MODULE 3 PART B: MAIZE PLOT INFORMATION: SOIL FERTILITY INDICATORS, CONSERVATION AGRICULTURE PRACTICES, AND CROP RESIDUE UTILIZATION

# {(1=Short rain season (Aug/Sept 2018 planting); 2= Long rain season (Feb/Marchl 2019 planting,3= current season (Aug/Sept 2019 planting))}

| Serial No |  | Parcel ID (start with one next to resi- dence) | plot ID | Plot lo- cation name | What is sur- rounding this plot | | Planting method 1.Row  2. Broad- casting | Soil Questions | | | Crop Rotation | Does the sub- plot suffer from soil loss? 1=yes; 0=No | Soil & water conserva- tion methods on this plot  (List 3 main tech- niques)  CODE 6 | | | Plot irri- gated 0.No  1.Yes |
| --- | --- | --- | --- | --- | --- | --- | --- | --- | --- | --- | --- | --- | --- | --- | --- | --- |
|  | Season  1=Short rain sea- son (Aug/Sept 2018 planting) 2=Long rain sea- son (Feb/March 2019 planting) 3=current season (Aug/Sept 2019  planting) |  |  |  |  |  |  | How fer- tile is the soil of this  [sub-  plot]? CODE 3 | What is the soil slope of this [sub- plot]?  CODE 4 | What is the soil depth of this [sub-  plot]? CODE 5 | Main crop grown during 2018  CODE 2 |  |  |  |  |  |
| 3B1 |  | 3B2 | 3B3 | 3B4a | 3B4b | 3B4c | 3B5 | 3B6a | 3B5b | 3B5c | 3B6 | 3B7 | 3B8a | 3B8b | 3B8c | 3B9 |
|  |  |  |  |  |  |  |  |  |  |  |  |  |  |  |  |  |
|  |  |  |  |  |  |  |  |  |  |  |  |  |  |  |  |  |

| CODE 2 | | | | CODE 3 | CODE 4 | CODE 5 | CODE 6 |
| --- | --- | --- | --- | --- | --- | --- | --- |
| Maize Sorghum Barley Millet Beans Soybeans Bananas  Sweet potato cassava | Groundnut Tomato Cabbage Black Paper Sesame | Garlic desmodium Mango Sugar Cane Coffee cucumber Pumpkins Watermelons Egg plants Okra | Eucalyptus Grazing land Fallow land Haricot bean  Other crop, specify... | 1. Good 2. Medium 3.Low | 1. Gentle slope (flat) 2. Medium slope 3. Steep slope | 1. Shallow 2. Medium 3.Deep | 1. None 5.Minimum/zero 2. Terraces till 3. Mulching 6.Soil bunds 4. Grass strips 7.Stone bunds 5. Trees on bound- 8.Box ridges aries 9.Other, spec-   ify……. |

MODULE 3 PART C: Utilization of chemicals, Manure and fertilizer on maize plot for: {1=Short rain season (Aug/Sept 2018 planting); 2= Long rain season (Feb/March 2019 planting,3= current season (Aug/Sept 2019 planting))}

|  | Season  1=Short rain season (Aug/Sept 2018 planting)  2=Long rain season (Feb/March 2019 planting)  3=current sea- son (Aug/Sept 2019 planting) | | Parcel ID (Same order as in above) | Plot ID  (Same order as in above) | Insecticides use | | | | Herbicides | | | | | Fertilizer use on this[sub-plot] | | | | | | | Manure use |
| --- | --- | --- | --- | --- | --- | --- | --- | --- | --- | --- | --- | --- | --- | --- | --- | --- | --- | --- | --- | --- | --- |
|  |  |  |  |  | Has this plot re- ceived insecti- cides?  1= Yes 0=No | Type of insec- ticides Codes to be created | Total quantity used  (kg or lit- ter) | Total cost (UGX) | Has this plot received herb- icides?  1=Yes 0=No | Type of herbi- cides? Codes to be created | | Quantity of herbicide used on this [sub- PLOT]  (kg or lit- ter) | Total cost (UGX) | Has this [Sub-plot] received UREA  1=Yes>>;  0=No | Quantity (kg) | Total cost (UGX) | Has this [Sub-plot] received DAP  1=Yes 0=No | Quantity (kg) | Total cost (UGX) | | Has this [Sub- plot] re- ceived Manure  1=Yes; 0=No |
|  | 3C2 | | 3C3 | 3C4 | 3C5a | 3C5b | 3C5c | 3C5d | 3C6a | 3C6b | | 3C6c | 3C6d | 3C7a | 3C7b | 3C7c | 3C7d | 3C7e | 3C7f | | 3C8 |
|  |  | |  |  |  |  |  |  |  |  | |  |  |  |  |  |  |  |  | |  |
|  |  | |  |  |  |  |  |  |  |  | |  |  |  |  |  |  |  |  | |  |
|  |  | |  |  |  |  |  |  |  |  | |  |  |  |  |  |  |  |  | |  |
|  | | Code 7 | | | | | | | | | Code 8 | | | | | | | | |  | |
|  |  | Rocket striker amadox Dudumac Duduciper  Tafuga | | | | | | | | |  | | | | | | | | |  |  |

MODULE 3 PART D: MAIZE Plot information: LABOUR FOR THE THREE MAIN CROP PRODUCTION ACTIVITIES FOR: {1=Short rain season (Aug/Sept 2018 planting); 2= Long rain season (Feb/March 2019 planting,3= current season (Aug/Sept 2019 planting))}

| Serial No |  | Parcel ID (Same order as in above) | Plot ID  (Same order as in | Seed use | | | | Ploughing and planting | | | | | | Weeding | | |
| --- | --- | --- | --- | --- | --- | --- | --- | --- | --- | --- | --- | --- | --- | --- | --- | --- |
|  | Season 1=Short rain sea- son  (Aug/Sept |  |  | Quan- tity of non- bough t | Quan- tity of bought seed | Total cost of bough t seed | Type of seed used Local | What did you use for | How many times was this [sub- | If  *3D5a=*  *1* | If  *3D5a=*  *2*  Total cost of | Total fam- ily labor in person days (ploughing & planting) | Total hired labor in per- son days (ploughing & planting) | How many times was this | Total family labor in person days  - no. of | Total hired labor in per- son days |

|  | 2018  planting) 2=Long rain sea- son (Feb/Mar ch 2019 planting) 3=current season (Aug/Sept 2019  planting) |  |  | seed (kg (own saved, farm- ers to farm- ers ex- chang e, etc.) | (in- clud- ing seed bought using credit) (kg/no  .) | (UGX  ) (in- clud- ing seed bough t for credit  ) | Im- prove d Both | ploug hing? 1.Ani- mal trac- tion 2.Trac tor 3.Han d hoe | PLOT]  ploughe d in- cluding planting | Total cost of hired oxen (UGX) | hired tractor (UGX ) | | –no. of ef- fective days used | |  | | [sub- PLOT]  weeded  ? | people in adult equivalent X no. of effective days used | | (if no hired labor put zero) | |
| --- | --- | --- | --- | --- | --- | --- | --- | --- | --- | --- | --- | --- | --- | --- | --- | --- | --- | --- | --- | --- | --- |
|  |  |  |  |  |  |  |  |  |  |  |  |  | mal e | fe- male | mal e | fe- mal e |  | Mal e | fe- mal e | male | fe- mal e |
| 3D  1 |  | 3D  2 | 3D3 | 3D4a | 3D4b | 3D4c | 3D4d | 3D5a | 3D5b |  |  | 3D5c | | 3D5  d | 3D5  e | 3D5  f | 3D6a | 3D6  b | 3D6  c | 3D6  d | 3D6  e |
|  |  |  |  |  |  |  |  |  |  |  |  |  | |  |  |  |  |  |  |  |  |
|  |  |  |  |  |  |  |  |  |  |  |  |  | |  |  |  |  |  |  |  |  |
|  |  |  |  |  |  |  |  |  |  |  |  |  | |  |  |  |  |  |  |  |  |

MODULE 3 PART D (CONT’D): Plot information: LABOUR FOR THE THREE MAIN CROP PRODUCTION ACTIVITIES FOR THE SEASONS: {1=Short rain season (Aug/Sept

# 2018 planting); 2= Long rain season (Feb/March 2019 planting)}}

| Serial No |  | Parcel ID (Same order as in above) | Plot ID  (Same order as in above) | Harvesting | | | | Threshing | | | | Total cost of hired labor (UGX) |
| --- | --- | --- | --- | --- | --- | --- | --- | --- | --- | --- | --- | --- |
|  | 1=Short rain season (Aug/Sept 2018 plant- ing) 2=Long rain season (Feb/March 2019 plant- ing) |  |  | Total family labor in person days | | Total hired labor in person days | | Total family labor in person days | | Total hired labor in person days | |  |
|  |  |  |  | male | female | male | female | male | female | male | female |  |
| 3D1 |  | 3D2 | 3D3 | 3D7a | 3D7b | 3D7c | 3D7d | 3D8a | 3D8b | 3D8c | 3D8d | 3D9 |
|  |  |  |  |  |  |  |  |  |  |  |  |  |
|  |  |  |  |  |  |  |  |  |  |  |  |  |
|  |  |  |  |  |  |  |  |  |  |  |  |  |

MODULE 3 PART E: PLOT INFORMATION: PRODUCTION STRESS, AND CROPS HARVESTED: : {1=Short rain season (Aug/Sept 2018 planting); 2= Long rain season (Feb/March 2019 planting}

| Serial No | 1=Short rain sea- son (Aug/Sept 2018  planting) 2=Long rain sea- son (Feb/Marc h 2019  planting)  ) | Parcel ID (Same order as in above) | Plot ID  (Same order as in above) | (this in the software is prepared to all intercropped crops (if intercropping)  NOTE CSPRO Programmer: Allow for three answer for strategies for FAW, Stemborer and Striga | | | | | | | | | | | | other major other stresses maize suffered from CODE 2 | | | Total pro-  duc- tion |
| --- | --- | --- | --- | --- | --- | --- | --- | --- | --- | --- | --- | --- | --- | --- | --- | --- | --- | --- | --- |
|  |  |  |  | Did crop suffer from Fall armywor m (FAW)?  1=Yes; 0=No | % of pro- duction lost due to fall armywor m | Strategies used to con- trol for FAW infestation | | Did crop suffer from stem- borer (FAW)  ?  1=Yes; 0=No | % of crop pro- duc- tion lost due to stem  -  bore r | Strategies used to con- trol for stem- borers infes-  tation | | Did crop suffer from Striga [sub- PLOT]  ?  1.Yes 0.NO | % of crop pro- duc- tion lost due to Strig a | Strategy to control for Striga | | Did this plot suf- fer from any other stres s e.g. dis- ease (suc h as (e.g. ear rot,  rust,) | Stress 1 | Stress 2 | har- veste d (dry  +  fresh- dry equiv alent) in kg |
|  |  |  |  |  |  | Strat egy 1  code 3 | Strat egy 2  code 3 |  |  | Strat egy 1  code 3 | Strat egy 2  code 3 |  |  | Strat- egy 1 code 3 | Strat egy 2  code 3 |  |  |  |  |
| 3E  1 |  | 3E  2 | 3E3 | 3E4a | 3E4b | 3E5a | 3E5  b | 3E6 | 3E7 | 3E8a | 3E8  b | 3E9 | 3E10 | 3E11  a | 3E1  b | 3E1  2 | 3E13a | 3E13b | 3E14 |
|  |  |  |  |  |  |  |  |  |  |  |  |  |  |  |  |  |  |  |  |
|  |  |  |  |  |  |  |  |  |  |  |  |  |  |  |  |  |  |  |  |

| CODE 2 | | | CODE 3 |
| --- | --- | --- | --- |
| 1. No Stress 2. Soil loss 3. Striga (show pic) 4. other Insects/pests 5.Disease | 5.WaterLogging 6.Drought 7.Frost  8. Flood | 9.Hailstorm 10.Animal trampling  11.Other, specify……………. | 1= Malathion  2=Diznon 60 3=crop rotation 4= intercropping  5= manure application  6= DDT  7= Early planting  8= Ash/pepper  9= herbal plants  10= Over weeding/ Frequent weed- ing  11=Uprooting infested plant and burning  12= Pesticides application  13= mechanically remove the insect pest |

|  |  |  | 14= Improved varieties (Kayongo- Go) |
| --- | --- | --- | --- |

MODULE 3 PART X: What support have you or any member of this household received on FAW, Striga and stemborer who provided the support?

D1. Have your or any member of this household received on any kind of support on FAW, Striga and stem borer? (1=yes>>>D2, 0-No) If yes, what kind of support have you or any member of your household received on FAW, Striga and stem borer and who provided the support?

| Support received for FAW (code 1) | Institutions that provided the support to the household (code 2) |
| --- | --- |
|  |  |
|  |  |
|  |  |
| Support received on Striga (code 1) |  |
|  |  |
|  |  |
|  |  |
| Support received for Stemborer (code 1) |  |
|  |  |
|  |  |
|  |  |
|  |  |

| Code 1: | Code2 |
| --- | --- |
| information and advisory | 1=Sasakwa global 2000 |
| training on proper management | 2=One-acre fund |
| pesticides | 3=NAADs/Local Government |
| Improved seed | 4=Food for the hungry |
| Others (specify) | 5=Others (specify) |

| 3A8 | 3A9 | 3A10 | 3A11 | 3A12 |
| --- | --- | --- | --- | --- |

Module 3 part F : DECISIONS ON MAIZE PRODUCTION/OUTPUT (ONLY MAIZE)

| 1=Short rain |  |  |  | Who |  |  | Total value |
| --- | --- | --- | --- | --- | --- | --- | --- |
| season |  |  | Who in | made de- |  |  | (E6 x E7 |
| (Aug/Sept |  |  | the house- | cisions |  |  |  |
| 2018 plant- ing) 2=Long rain  season | Market sold Codes A | Who in the household sold [Crop}?  Code C | hold make the deci- sion to  sale | over in- come from  maize | Quantity sold (Kg) | Average price (UGX )  /kg) |  |
| (Feb/March |  |  | [crop]? | sales? |  |  |  |
| 2019 plant- |  |  | Code C | Code C |  |  |  |
| ing) |  |  |  |  |  |  |  |
| 3F1 | 3F2 | 3F3 |  |  | 3E 5 | 3E 6 | 3E 6 |
|  |  |  |  |  |  |  |  |

| Codes A   1. Farm gate 2. Village market 3. Main/district market | Codes B 4. Donkey   1. Bicycle 5. Oxen/horse/donkey cart 2. Hired truck 6. Back/head load 3. Public transport 7. Other, specify…. | Code C 5=head and other household member(s)  1=head 6=Spouse and other household member(s)  2=Spouse 7=head, spouse and other household member(s) 3=head and spouse jointly  4=Other household member  5=head and other household member(s) 6=Spouse and other household member(s) 7=head, spouse and other household mem-  ber(s) |
| --- | --- | --- |

MODULE 3G: HOUSEHOLD INCOME from crops and nonfarm activities Income sources and income earned in the past twelve months

| Income source | Income source codes | Did the household earn income from [income source] past twelve months?  1 = Yes, 2 = No | How much did the household earn in Cash (UGX) from [income source] dur- ing the past twelve months  (UGX) | How much did the households receive In-kind (esti- mated cash equivalent in (UGX) from [income source] during the past twelve months? | Total income |
| --- | --- | --- | --- | --- | --- |
| s/n | 3G1 | 3G2 | 3G3 | 3G4 | 3G 5 |
| sorghum | 2 |  |  |  |  |
| Millet | 3 |  |  |  |  |
| Cassava | 4 |  |  |  |  |
| Soya beans sales | 5 |  |  |  |  |
| Sunflower sales | 6 |  |  |  |  |
| Cotton sales | 7 |  |  |  |  |
| Groundnuts sales | 8 |  |  |  |  |
| Tobacco sales | 9 |  |  |  |  |
| Fruits sales | 10 |  |  |  |  |
| Cotton |  |  |  |  |  |
| Bananas |  |  |  |  |  |
| Sugar cane |  |  |  |  |  |
| coffee |  |  |  |  |  |
| Vegetable sales | 11 |  |  |  |  |
| Other crop (specify) | 12 |  |  |  |  |
| Salaried employment | 22 |  |  |  |  |
| Shop | 23 |  |  |  |  |
| Salon | 24 |  |  |  |  |
| Grain milling | 25 |  |  |  |  |
| Grain trading | 26 |  |  |  |  |
| Other non-agricultural business | 27 |  |  |  |  |
| Casual labor on farm (on-farm and off farm) | 28 |  |  |  |  |
| Casual labor off farm | 29 |  |  |  |  |
| Selling charcoal | 30 |  |  |  |  |
| selling firewood | 31 |  |  |  |  |
| Brick making | 32 |  |  |  |  |
| Transportation busi- nesses | 33 |  |  |  |  |
| Other income source (specify) | 34 |  |  |  |  |

MODULE 4: LIVESTOCK OWNERSHIP AND PRODUCTION

PART A: LIVESTOCK OWNERSHIP, MARKETING AND PRODUCTION COSTS IN THE LAST 12 MONTHS

4A1. Is fodder a major constraint to livestock production in your household? 1=yes; 0=no 4A2. Did your household purchase fodder in the last 12 months? 1=yes; 0=no

4A3. If yes to question 4A2, what type of fodder did you buy? 1= hay; 2= oil by-products; 3=other specific

4A4. If yes to question 4A2, how much did you spend on fodder in the past 12 months? (UGX) Livestock inventory

| No | Animal type | Does the house- hold own […] 0=No; 1=Yes | No. owned | Value of each if sold today UGX | Production and Marketing | | |
| --- | --- | --- | --- | --- | --- | --- | --- |
|  |  |  |  |  | Did the hhld sell  […]?  0=No; 1=Yes | Quantity sold (Number  /kg/lit) | Average selling price UGX |
|  |  |  | Total owned by Household |  |  |  |  |
|  | 4A5 | 4A6 | 4A7 | 4A8 | 4A9 | 4A10 | 4A11 |
| 1 | Indigenous cows |  |  |  |  |  |  |
| 2 | Cross bred  /exotic cow |  |  |  |  |  |  |
| 3 | Oxen |  |  |  |  |  |  |
| 4 | Bulls |  |  |  |  |  |  |
| 5 | Heifers |  |  |  |  |  |  |
| 6 | Calves |  |  |  |  |  |  |
| 7 | Small livestock (goats + sheep) |  |  |  |  |  |  |
| 8 | Pig |  |  |  |  |  |  |
| 9 | Donkeys |  |  |  |  |  |  |
| 10 | Horse |  |  |  |  |  |  |
| 11 | Mule |  |  |  |  |  |  |
| 12 | Poultry (local chicken, improved chicken, ducks, etc.) |  |  |  |  |  |  |

PART A(Continued): LIVESTOCK PRODUCTION COSTS IN THE LAST 12 MONTHS

| s/n |  | Did you spend on this item | If yes, how much (UGX) |
| --- | --- | --- | --- |
|  | 4A12 | 4A13 | 4A14 |
|  | Fodder/forage including ha |  |  |
|  | Labor |  |  |
|  | Veterinary care |  |  |
|  | Artificial insemination |  |  |
|  | slat |  |  |
|  |  |  |  |

MODULE 4PART B: LIVESTOCK OUTPUT AND LIVESTOCK FATTENING PRACTICES IN THE LAST 12 MONTHS

PARTB1: MILKING COWS: Does your household have milking cows? 1=yes 0=no

| s/n | milking cows’  type  1= improved; 0=local | number of milking cows | calving inter- val (year) | Average lacta- tion length months) | Total amount of milk in 2018/19(li- ter) | Who milks the cows?  Code C | Who sells the milk?  Code C | Who uses income from milk sale? Code C | Who consumes milk?  Code C |
| --- | --- | --- | --- | --- | --- | --- | --- | --- | --- |
|  | 4B1 | 4B2 | 4B3 | 4B4 | 4B5 | 4B6 | 4B7 | 4B8 | 4B9 |
|  |  |  |  |  |  |  |  |  |  |
|  |  |  |  |  |  |  |  |  |  |

1. Spouse and other female family member(s)
2. Head and other male + female family members
3. Head, spouse and other male family member(s)
4. Head spouse and other female family member(s)
5. Head, spouse and other male +female family member(s)
6. children

CODE C

1. Head
2. Spouse
3. Head and spouse jointly
4. Head and other male family member(s)
5. Head and other female family member(s)
6. Spouse and other male family member(s)

PART B2. Livestock products and utilization during in the last 12 months

| Product | Did house- hold pro- duce [Prod-  uct]? 1=yes; 0=n0 | Unit (lit/kg/no) | Amount produced | Quantity consumed home | Quantity sold | Average price UGX | Total value sold  [8B15x 8B16] | Quantity used for other pur- pose (kg) | Did you buy [prod- uct]?  1=yes; 0=no | If yes, amount pur- chased | Unit (lit/kg/no) | Value of purchased (UGX) |
| --- | --- | --- | --- | --- | --- | --- | --- | --- | --- | --- | --- | --- |
| 4B10 | 4B11 | 4B12 | 4B13 | 4B14 | 4B15 | 4B16 | 4B17 | 4B17 | 4B18 | 4B19 | 4B20 | 4B21 |
| Eggs |  |  |  |  |  |  |  |  |  |  |  |  |
| Bong |  |  |  |  |  |  |  |  |  |  |  |  |
| Ghee/butter |  |  |  |  |  |  |  |  |  |  |  |  |
| Other livestock  products (honey, fish) |  |  |  |  |  |  |  |  |  |  |  |  |
| hides/skins |  |  |  |  |  |  |  |  |  |  |  |  |
| butter/cheese |  |  |  |  |  |  |  |  |  |  |  |  |

| milk/cream |  |  |  |  |  |  |  |  |  |  |  |  |
| --- | --- | --- | --- | --- | --- | --- | --- | --- | --- | --- | --- | --- |
| dung cakes |  |  |  |  |  |  |  |  |  |  |  |  |
|  |  |  |  |  |  |  |  |  |  |  |  |  |
|  |  |  |  |  |  |  |  |  |  |  |  |  |

1. Spouse and other female family member(s)
2. Head and other male + female family members
3. Head, spouse and other male family member(s)
4. Head spouse and other female family member(s)
5. Head, spouse and other male +female family member(s)
6. children

CODE C

1. Head
2. Spouse
3. Head and spouse jointly
4. Head and other male family member(s)
5. Head and other female family member(s)
6. Spouse and other male family member(s)

Module 5. Social Capital and Access to Information

| No. | Question (*Instructions*) | | Response |
| --- | --- | --- | --- |
| 5A1 | How many years have you been living in this village? (in years) | Primary male | Years |
|  |  | Primary female | Years |
| 9A2 | In the last 12 months, did you have a need for advice or information from an agricultural extension officer? No 1. Yes | Primary male |  |
|  |  | Primary female |  |
| 5A3 | In the last 12 months, did you receive any advice or information from an agricultural extension officer? No 1. Yes | Primary male |  |
|  |  | Primary female |  |
| 5A4 | How long does it take, in minutes, to walk (one way) from your residence to the place where you typically receive agricultural extension advice? | | Minutes (one way) |
| 5A5 | Are you confident of the skills and knowledge of extension officers to provide extension advice? No 1. Yes | |  |
| 5A6 | Did you or any other household member have a need for credit in the last 12 months? | Primary male |  |
|  |  | Primary female |  |
| 5A7 | Did you receive credit from any source in the last 12 months? | Primary male |  |
|  |  | Primary female |  |
| 5A8 | Do you think you can rely on government support (e.g., subsidies, food aid) if your crop fails? No 1. Yes | |  |
| 5A9 | How many people within and outside your village can you rely on in times of critical needs? | | people |

MODULE 6: HOUSEHOLD AND INDVIDUAL FOOD SECURITY AND DIEATRY DIVERSTY SCORE MODULE 6 PART A. DIETARY DIVERSITY SCORE

10A1 How is the consumption habit in the household? Codes: 1=Spouses eat together same type of food all time; 2=Husband given priority to eat first food

prepared for household; 3=Wife eats what is remaining after other hhld members ate; 4=Children given priority to eat first food prepared for household; 5=All household members including children eat same type of food at all time

Now I would like to ask you the types of foods (meals and snack) children and mother ate or drank yesterday during day and night, whether at home or outside the home. Please, tell me by accurately recalling.

(THIS QUESTION MUST BE ADMINSTRED IN THE PRESENCES OF WOMEN (WIFE OR WOMEN IN CHARGE OF COOKING FOOD)

|  |  |  | Did they eat or drink any of [Food]? | | | |
| --- | --- | --- | --- | --- | --- | --- |
|  |  |  | Child/children (up to 59 months) | | Mother to the child/children | |
| Sr.No. | 6A2 | 6A3 | 6A4 | 6A5 | 6A6 | 6A7 |
| G11.01  Code | G11.01  Food group | G11.01  Description | Yesterday (day & night) (1=Yes;  0=No) | 7 days (1=Yes; 0=No) | Yesterday (day & night) (1=Yes;  0=No) | 7 days (1=Yes; 0=No) |
|  | CEREALS | Corn/maize, rice, barley, oats, wheat, sorghum, finger millet or any other grains or foods made from these (e.g. bread, biscuits, noodles, porridge, or other grain products) |  |  |  |  |
|  | VITAMIN A RICH VEGETABLES AND TUBERS | Pumpkin, carrots, squash, or yellow/orange flesh sweet potatoes or *other locally available vitamin-A rich vegetables (e.g. red sweet pepper)* |  |  |  |  |
|  | WHITE TUBERS AND ROOTS | White potatoes, white yams, white cassava, plantains or other foods made from roots |  |  |  |  |
|  | DARK GREEN LEAFY VEGETA- BLES | Dark green/leafy vegetables, including wild ones + *lo- cally available vitamin-A rich leaves such as ama-*  *ranth, Cassava leaves, Kale, Spinach,* Broccoli, Pumpkin greenest |  |  |  |  |
|  | OTHER VEGETA- BLES | Other vegetables (e.g. tomato, onion, eggplant, Cauli- flower, Cabbage (common and red varieties, Cucum- bers , eggplants, Mushroom , Onion , okra, Green pepper , Peas, green, when eaten as fresh pod , To-  mato , mock tomato ,green maize, Beans (various) when eaten as fresh pods, Beets) , wild vegetables |  |  |  |  |
|  | VITAMIN A RICH FRUITS | Ripe mangoes, cantaloupe, apricots (fresh or dried passion fruits, peaches, Ripe papaya (ripe, fresh and  dried),, dried peaches + *other locally available vita- min A-rich fruits* |  |  |  |  |
|  | OTHER FRUITS | Other fruits, including wild fruits (e.g. Coconut flesh, bananas, avocado, apple, lemon, lime, orange, pineap- ple, Watermelon, Tangerine, Cherries, Jackfruit, guava, Pineapple, pears, Strawberry, plums, |  |  |  |  |

|  |  | Pomegranate, grapes/grape fruits, Blueberry, black-  berry, black current… ) |  |  |  |  |
| --- | --- | --- | --- | --- | --- | --- |
|  | ORGAN MEAT / (IRON RICH) | Liver, Kidney, Heart or other organ meats or blood-  based foods (e.g ) |  |  |  |  |
|  | FLESH MEATS | Beef, pork, lamb, goat, wild game, chicken, or other birds |  |  |  |  |
|  | EGGS | Chicken, duck, guinea hen or any other egg |  |  |  |  |
|  | FISH | Fresh or dried fish or shellfish |  |  |  |  |
|  | PULSES (BEANS, PEAS & LENTILS) | Mature beans or peas (fresh or dried seed), lentils or their products, |  |  |  |  |
|  | NUTS AND SEEDS | Groundnuts, tree nuts, other nuts, sesame, seeds or foods made from these |  |  |  |  |
|  | MILK AND MILK PRODUCTS | Milk, cheese, yogurt or other milk products but NOT including butter, ice cream, cream or sour cream |  |  |  |  |
|  | OILS AND FATS | Oil, fats or butter added to food or used for cooking, oil seeds and foods made from oil seeds e.g. sun- flower |  |  |  |  |
|  | RED PALM PROD- UCTS | Red palm oil, palm nut or palm nut pulp sauce |  |  |  |  |
|  | SWEETS | Sugar, honey, sweetened soda or sugary foods such as chocolates, candies, cookies and cakes |  |  |  |  |
|  | SUGAR-SWEET- ENED BEVERAGES | Sweetened fruit juices and “juice drinks”, soft drinks/fizzy drinks, chocolate drinks, malt drinks, yo- ghurt drinks or sweet tea or coffee with sugar |  |  |  |  |
|  | CONDIMENTS AND SEASONINGS | Ingredients used in small quantities for flavor such as Spices (black pepper, salt), chilies, herbs, condiments (soy sauce, hot sauce), royco, tomato paste etc |  |  |  |  |
|  | OTHER BEVER- AGES | Tea or coffee if not sweetened, clear broth, alcohol |  |  |  |  |
|  | SAVOURY AND FRIED SNACKS | Crisps and chips, fried dough or other fried snacks |  |  |  |  |
|  | MEAL OUTSIDE HOME | Meal or snack outside of the home |  |  |  |  |

MODULE 6 PART B: FOR EACH OF THE FOLLOWING QUESTIONS, PLEASE CONSIDER WHAT HAS HAPPENED IN THE PAST 12 MONTHS

| s/n | Question | Response code | Response options (Codes) |
| --- | --- | --- | --- |
|  | 6B1 | 6B2 | 6B3 |
| 1. | What is your own assessment of the adequacy of your family’s food consumption over the  past 12 months? |  | 1=It was less than adequate for your family’s needs  2=It was just adequate for your family’s needs  3=It was more than adequate for your family’s needs  4=Not applicable  ‘‘Adequate’’ means no more nor less than what the respondent  considers to be the minimum consumption needs of the family |
| 2. | What is your own assessment of the adequacy of your family’s housing over the past 12  months? |  |  |
| 3. | What is your own assessment of the adequacy of your family’s clothing over the past 12  months |  |  |

| 4. | What is your own assessment of the adequacy of the health care your family gets over the past 12 months? |  |  |
| --- | --- | --- | --- |
| 5. | What is your own assessment of the adequacy of your children’s schooling over the past  12 months? |  |  |

ANNEX 1: CROP CODES

| Maize Sorghum Teff Wheat Barley Millet Beans Chick Peas | Groundnut Potato Tomato Cabbage Black Paper Papaya Lentil Linseed Sesame | Garlic desmodium Khat Banana Mango Sugar Cane Coffee Gesho | Eucalyptus Grazing land Fallow land Haricot bean  Other crop, specify... |
| --- | --- | --- | --- |
