# Supplementary material for: Fall armyworm infestation, maize production and nutrition security: Evidence from Uganda
Source: PLoS One. 2025 Dec 4;20(12):e0336785. doi: 10.1371/journal.pone.0336785 (PMC12677496; doi:10.1371/journal.pone.0336785)
Supplement: Attachment — Submitted filename: Responses to reviewers.docx [file pone.0336785.s005.docx]

**Responses to comments**

|  | **Comment** | **Action/response** |
| --- | --- | --- |
| 1. | Please ensure that your manuscript meets PLOS ONE's style requirements, including those for file naming. The PLOS ONE style templates can be found at  https://journals.plos.org/plosone/s/file?id=wjVg/PLOSOne_formatting_sample_main_body.pdf and  <https://journals.plos.org/plosone/s/file?id=ba62/PLOSOne_formatting_sample_title_authors_affiliations.pdf> | The manuscript has been significantly revised and re-formatted in line with the journal requirements. The revised /manuscript has been re-submitted through the PLOS ONE online system |
| **2.** | Please include a complete copy of PLOS’ questionnaire on inclusivity in global research in your revised manuscript. Our policy for research in this area aims to improve transparency in the reporting of research performed outside of researchers’ own country or community. The policy applies to researchers who have travelled to a different country to conduct research, research with Indigenous populations or their lands, and research on cultural artefacts. The questionnaire can also be requested at the journal’s discretion for any other submissions, even if these conditions are not met.  Please find more information on the policy and a link to download a blank copy of the questionnaire here: https://journals.plos.org/plosone/s/best-practices-in-research-reporting. Please upload a completed version of your questionnaire as Supporting Information when you resubmit your manuscript. | We have included the filled-in copy of PLOS’ questionnaire on inclusivity in global research as part of the supporting documents. We have also included our data collection questionnaire in the supporting documents. |
| **3.** | Please provide additional details regarding participant consent. In the ethics statement in the Methods and online submission information, please ensure that you have specified (1) whether consent was informed and (2) what type you obtained (for instance, written or verbal, and if verbal, how it was documented and witnessed). If your study included minors, state whether you obtained consent from parents or guardians. If the need for consent was waived by the ethics committee, please include this information. | We have stated that consent was obtained verbally from respondents. A statement was included in the questionnaire and read out by enumerators to each of the respondents before the interview proceeds. |
|  | If you are reporting a retrospective study of medical records or archived samples, please ensure that you have discussed whether all data were fully anonymized before you accessed them and/or whether the IRB or ethics committee waived the requirement for informed consent. If patients provided informed written consent to have data from their medical records used in research, please include this information. | N/A |
| **4.** | We note that the grant information you provided in the ‘Funding Information’ and ‘Financial Disclosure’ sections do not match.  When you resubmit, please ensure that you provide the correct grant numbers for the awards you received for your study in the ‘Funding Information’ section. | Thanks. The correct grant information is: European Union (DCI-FOOD/2018/402-634) |
| **5.** | Thank you for stating the following financial disclosure:  “financial support for this research by the following organizations and agencies: European Union (DCI-FOOD/2018/402-634)”  Please state what role the funders took in the study.  If the funders had no role, please state: "The funders had no role in study design, data collection and analysis, decision to publish, or preparation of the manuscript."  If this statement is not correct you must amend it as needed.  Please include this amended Role of Funder statement in your cover letter; we will change the online submission form on your behalf. | Yes, the funders only supported the study financially. “Funders had no role in study design, data collection and analysis, decision to publish, or preparation of the manuscript” |
| **6.** | We note that Figure A2 in your submission contain map images which may be copyrighted. All PLOS content is published under the Creative Commons Attribution License (CC BY 4.0), which means that the manuscript, images, and Supporting Information files will be freely available online, and any third party is permitted to access, download, copy, distribute, and use these materials in any way, even commercially, with proper attribution. For these reasons, we cannot publish previously copyrighted maps or satellite images created using proprietary data, such as Google software (Google Maps, Street View, and Earth). For more information, see our copyright guidelines: http://journals.plos.org/plosone/s/licenses-and-copyright.  We require you to either (1) present written permission from the copyright holder to publish these figures specifically under the CC BY 4.0 license, or (2) remove the figures from your submission: | I have removed the figure from the submission |
